# Preferences for Hypertension Care in Malawi: A Discrete Choice Experiment Among People Living with Hypertension, With and Without HIV

**DOI:** 10.1007/s10461-024-04492-y

**Published:** 2024-09-13

**Authors:** Risa Hoffman, Khumbo Phiri, Pericles Kalande, Hannah Whitehead, Agnes Moses, Peter C. Rockers, Chi-Hong Tseng, George Talama, Jonathan Chiwanda Banda, Joep J. van Oosterhout, Sam Phiri, Corrina Moucheraud

**Affiliations:** 1 David Geffen School of Medicine, Department of Medicine, Division of Infectious Diseases, University of California, Risa Hoffman, 911 Broxton Avenue Suite 301D, Los Angeles, CA 90024 USA; 2 Partners in Hope Medical Center, Lilongwe, Malawi; 3 Department of Global Health, Boston University School of Public Health, Boston, MA USA; 4 David Geffen School of Medicine, Department of Medicine, Division of General Internal Medicine and Health Services Research, University of California, Los Angeles, CA USA; 5 Non-Communicable Disease and Mental Health Division, Ministry of Health, Lilongwe, Malawi; 6 School of Global and Public Health, Kamuzu University of Health Sciences, Lilongwe, Malawi; 7 Department of Public Health Policy and Management, New York University Global School of Public Health, New York, NY USA

**Keywords:** HIV, Hypertension, Client-centered care, Discrete choice experiment

## Abstract

Hypertension is the most common non-communicable disease diagnosed among people in sub-Saharan Africa. However, little is known about client preferences for hypertension care. We performed a discrete choice experiment in Malawi among people with hypertension, with and without HIV. Participants were asked to select between two care scenarios, each with six attributes: distance, waiting time, provider friendliness, individual or group care, antihypertensive medication supply, and antihypertensive medication dispensing frequency (three versus one month). Eight choice sets (each with two scenarios) were presented to each individual. Mixed effects logit models quantified preferences for each attribute. Estimated model coefficients were used to predict uptake of hypothetical models of care. Between July 2021 and April 2022 we enrolled 1003 adults from 14 facilities in Malawi; half were living with HIV and on ART for a median of 11 years. Median age of respondents was 57 years (IQR 49–63), 58.2% were female, and median duration on antihypertensive medications was 4 years (IQR 2–7). Participants strongly preferred seeing a provider alone versus in a group (OR 11.3, 95% CI 10.4–12.3), with stronger preference for individual care among those with HIV (OR 15.4 versus 8.6, p < 0.001). Three-month versus monthly dispensing was also strongly preferred (OR 4.2; 95% CI 3.9–4.5). 72% of respondents would choose group care if all other facility attributes were favorable, although PLHIV were less likely to make this trade-off (66% versus 77%). These findings have implications for the scale-up of hypertension care in Malawi and similar settings.

## Introduction

With the widespread scale-up of antiretroviral therapy (ART), people living with HIV (PLHIV) in resource-limited settings are living longer and developing chronic, non-communicable diseases (NCDs) of aging at rates similar to or greater than the general population [1, 2]. Multimorbidity in PLHIV poses unique challenges within constrained health systems given that resources for HIV care typically do not extend to NCDs [3]. While integrated HIV and NCD care models (in which care for all chronic conditions is provided by the same clinician at the same time) may offer a more client-centered approach by lowering the burden of care-seeking, few low-resource countries have adopted this approach [4, 5]. In Africa, PLHIV diagnosed with hypertension or other NCDs are often referred to outpatient departments (OPDs) or specialized NCD clinics, which adds time, cost, and logistical burdens. Additionally, while differentiated service delivery models for HIV care have advanced in Africa, including widespread multi-month dispensing of ART (dispensing either three or six months of ART for stable clients) [6] and community-based treatment delivery [7], these innovations have not been implemented for NCD care nor for PLHIV with NCD comorbidities. Further, there are limited data on how PLHIV would prefer to receive care for NCD comorbidities – but such information is essential to inform the development and evaluation of client-centered models of care.

Malawi has a significant double burden of HIV and hypertension, with an adult HIV prevalence of 8.9% [8] and hypertension prevalence of approximately 30% in the general population [9]. Hypertension prevalence in PLHIV has been reported in the range of 24%-46% [10, 11]. In 2016, Malawi’s national HIV treatment guidelines recommended annual blood pressure screening during HIV care [12]. While the goal for Malawi’s health system is to provide integrated HIV and NCD care for all PLHIV [13, 14], most care is provided through referral to an OPD or specialized NCD clinic. OPD and NCD clinics may be offered at the same facility (often on a different day) or may require referral to a different facility. This fragmented care increases time spent on care-seeking, costs of transportation, costs for care and medications (if not free), and other opportunity costs, such as lost wages [15]. Given the high burden of hypertension in Malawian PLHIV, we sought to understand preferences for how PLHIV would like to receive care for HIV and hypertension using a discrete choice experiment (DCE) and to compare preferences to those living with hypertension and without HIV. DCEs ask participants to make hypothetical trade-offs between choices, thereby elucidating their preferences—a particularly relevant exercise when not all preferences can be satisfied, e.g., health services in resource-limited health systems such as Malawi.

## Methods

### Study Setting

This study took place at 14 health facilities in Central and Southern Malawi, including 11 government facilities where all care and medications were provided for free and three Christian Health Association of Malawi (CHAM) facilities. Of the CHAM sites in this sample, all provided free HIV care, but only one provided antihypertensive medications and care for free; the other two charged a consultation fee for NCD care and had antihypertensive medications available for purchase. At the time of the study, five facilities (across government and CHAM sites) were providing integrated care for HIV and hypertension, while the remainder were referring clients to a different clinic for care (either at that facility or a distant site).

Data were collected between July 2021 and April 2022. At the time of this study, HIV guidelines included three-month ART dispensing for stable clients; however, since the start of the COVID-19 pandemic (early 2020 onwards), six-month ART dispensing was widely implemented in Malawi. New HIV clinical management guidelines released in January 2022 formally recommended six-month dispensing for stable ART clients [6]. At the time of data collection, there was no formal guidance for antihypertensive medication refill and/or dispensing frequency in Malawi.

### Study Population and Data Collection

Potential participants were recruited from waiting areas of NCD clinics, ART clinics, and OPDs. In order to assess whether PLHIV have different preferences for hypertension care, we purposively recruited half of the sample to be HIV negative or have unknown HIV status (never tested), based on self-report. Eligible participants were ≥ 18 years of age, self-reported a previous diagnosis of high blood pressure, had been on antihypertensive medication for at least six months total in the previous three years, and, if living with HIV, had been on ART for at least six of the previous twelve months.

Convenience sampling was used to enroll study participants by asking consecutive adults in the waiting area if they would be interested in the study. While their place was held in line, screening and study procedures were performed by a research assistant in a private area. Surveys were orally administered, with the research assistant asking the questions and selecting answers on a tablet. The survey included information on sociodemographic characteristics and experiences with hypertension care, including frequency of visits (and for those with HIV, frequency of visits for ART and other HIV-related care); time spent receiving care (from arriving at clinic to leaving clinic); out-of-pocket costs for hypertension care and medication; costs of transportation to clinic; comorbid diabetes and heart disease diagnoses; and for PLHIV, whether care was integrated. All costs were collected in local currency (Malawi kwacha) and converted to US dollars based on historical exchange rates for the time period of data collection. For self-reported economic status, we asked participants to indicate their position on a six-step ladder with the lowest step representing the poorest status (coded as 1), and the highest step representing the wealthiest status (coded as 6) [16, 17].

**Fig. 1:**
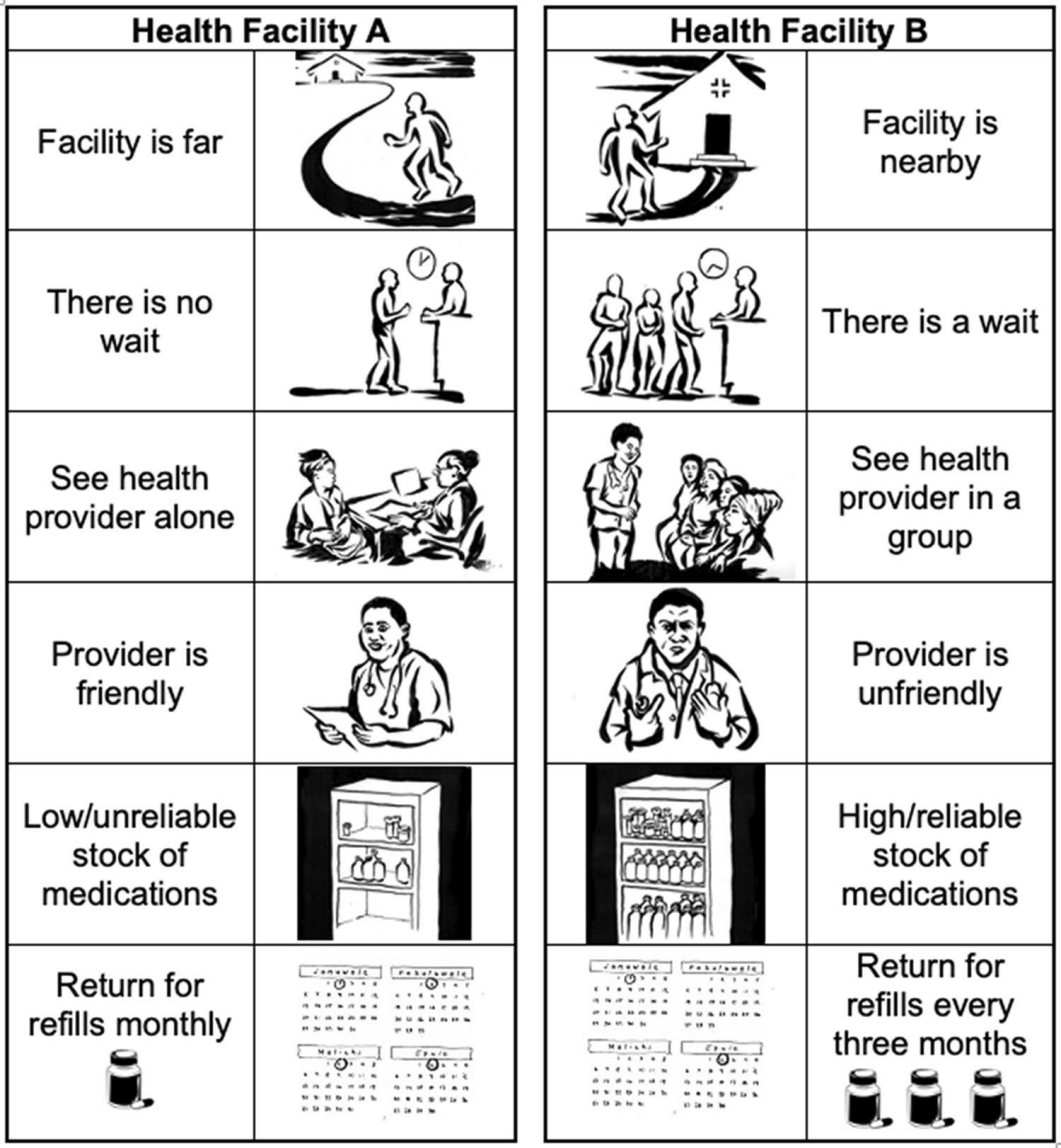
Sample choice task from DCE illustrating each attribute level. Deployed versions included Chichewa text instead of English

The brief survey was followed by administration of the DCE module, which elicited preferences regarding attributes of hypothetical models of hypertension care. Six attributes dichotomized at two levels were selected based on the qualitative results of our preliminary research with HIV and hypertension co-affected patients in Malawi [18]: (1) distance to the facility (near versus far), (2) wait time (short versus long), (3) frequency of facility visits for care and antihypertensive medication dispensing (three-monthly versus monthly), (4) friendliness of clinical staff (friendly versus unfriendly), (5) individual versus group care, and (6) hypertension medication supply reliability (high stock versus low stock) (Fig. 1).

Participants were instructed to make choices based on two conditions for all scenarios in the DCE: (1) all care and medications for hypertension would be provided for free; and (2) for PLHIV, their hypertension care would be delivered in an integrated model with a single clinician providing care for both HIV and hypertension, and medications would be given for both conditions during the same visit. These assumptions were made after consultation with the Malawi Ministry of Health [14], which aims to offer integrated care for PLHIV and to expand free access to care for hypertension in government facilities.

Ngene software [19] was used to generate an efficient, d-optimized design that maximized level balance, except for medication stock for which we included a slight attribute imbalance as we hypothesized this would be a dominating attribute. The final DCE design included 80 pairs of scenarios blocked into ten groups of eight choice sets. Respondents were randomized to one of the ten blocks within the survey and had to choose one of the two hypothetical models of care in each of the eight choice sets, with no opt-out option. Attributes, levels, and level balance are shown in Supplementary Table 1.

As this research was conducted among a low-literacy population, the DCE was presented in pictorial form with a distinct illustration for each attribute level (Fig. 1). The six facility attributes were explained to the respondent using a script that had been pre-tested for comprehension and clarity. Respondents took approximately 35 minutes to complete the entire survey (inclusive of DCE), on average.

After completion of the DCE, we performed a review of the client’s medical record for the most recent three blood pressure measurements in the prior year, blood pressure and ART medications in the prior year, and viral load measurements in the prior two years.

The survey was developed in English and translated into Chichewa, the local language, and back-translated to confirm that all questions retained their meaning and intent. All data were collected electronically using the SurveyCTO mobile data collection platform on Android tablets.

Respondents provided informed consent prior to commencement of study procedures. This research was approved by the Malawi National Health Science Research Committee (protocol number 20/07/2577) and the Institutional Review Board at the University of California, Los Angeles (protocol number 20-001856).

### Sample Size and Statistical Analysis

Our target sample size was 1000 individuals, 500 with hypertension and 500 with HIV and hypertension. There is no consensus about how to calculate DCE sample size based on statistical power [20, 21]. Our sample size is similar to previous DCE studies with a similar number of attributes and choice sets [22, 23].

Descriptive statistics and bivariate analyses (chi-squared tests, t-tests, Wilcoxon rank-sum tests) were calculated to describe and compare sociodemographic and clinical characteristics between respondents with and without HIV. We characterized the degree of blood pressure control overall and by HIV status using chart review data of the most recent two or three blood pressures in the prior year. If two blood pressures were available, control was defined as both < 140/90 mm Hg and if three were available, two of three were required to be < 140/90 mm Hg to be categorized as controlled. This threshold for control was chosen to align with the Malawi national treatment guidelines, which define blood pressure control as < 140/90 mm Hg [24]. Individuals with only one blood pressure measurement were excluded from this analysis.

For the DCE data, mixed effects logit models were used to estimate the direction and strength of preferences for each two-level attribute, and interaction models were used to assess differences in preferences by respondent demographics (gender, HIV status, age). All attribute variables were dummy coded. Estimated model coefficients were used to predict uptake of hypothetical models of hypertension care in five scenarios of interest that were selected based on the DCE results and expert input from co-investigators around realistic trade-offs that might be faced in Malawi based on feasible models for the delivery of hypertension care. Data cleaning and statistical analyses were conducted using Stata 17.0 [25] and DCE data were analyzed using R version 4.1.2 [26].

## Results

### Characteristics of Respondents

A total of 1003 respondents completed the survey and DCE; half (n = 501) were people living with both HIV and hypertension and half (n = 502) had hypertension only (Table 1). Approximately 60% of the respondents were female, with no difference in the gender distribution by HIV status; median age was 57 years (IQR 49–63); and PLHIV were an average of four years younger than those with hypertension only (54 versus 58 years). Nearly 90% of respondents were currently employed, and the majority (95%) self-identified as being in the bottom half of the wealth distribution. The majority (93%) of PLHIV were recruited from three urban facilities providing integrated care for HIV and hypertension.

**Table 1:** Sociodemographic and clinical characteristics of respondents, overall and by HIV status

	Hypertension N = 502	Hypertension and HIV N = 501	Overall N = 1003
Gender, N (%)			
Male	211 (42.0)	208 (41.5)	419 (41.8)
Female	291 (58.0)	293 (58.5)	584 (58.2)
Age			
Median (IQR) years	58 (51–66)	54 (49–60)	57 (49–63)
Marital Status, N (%)			
Single or no long-term partner	10 (2.0)	4 (0.8)	14 (1.4)
Married or long-term partner	379 (75.5)	331 (66.1)	710 (70.8)
Other (widowed, divorced, separated)	113 (22.5)	166 (33.1)	279 (27.8)
Employment, N (%)			
Working	447 (89.0)	448 (89.4)	895 (89.2)
Not working, looking	4 (0.8)	4 (0.8)	8 (0.8)
Not working or looking	51 (10.2)	49 (9.8)	100 (10.0)
Socioeconomic status (self-rated), N (%)			
Step 1 (poorest)	188 (37.4)	99 (19.8)	287 (28.6)
Step 2	181 (36.1)	191 (38.1)	372 (37.1)
Step 3	110 (21.9)	181 (36.1)	291 (29.0)
Step 4	19 (3.8)	30 (6.0)	49 (4.9)
Step 5	3 (0.6)	0 (0)	3 (0.3)
Step 6 (wealthiest)	1 (0.2)	0 (0)	1 (0.1)
Urban/Rural residence, N (%)			
Urban	344 (68.5)	343 (68.5)	687 (68.5)
Rural	158 (31.5)	158 (31.5)	316 (31.5)
Comorbidities (self-reported), N (%)			
Diabetes	121 (24.1)	45 (9.0)	166 (16.6)
Heart disease	20 (4.0)	10 (2.0)	30 (3.0)
Have taken antihypertensive medications on regular basis over past 30 days, N (%)	454 (90.4)	466 (93.0)	920 (91.7)
Median years with hypertension (IQR)	5 (2–8)	5 (3–8)	5 (3–8)
Median years on antihypertensive medication*	4 (2–7)	5 (3–7)	4 (2–7)
Antihypertensive medications, N (%)**			
Diuretic	141 (28.4)	222 (44.4)	363 (36.4)
Diuretic+CCB	97 (19.5)	95 (19.0)	192 (19.3)
Diuretic + CCB+ACEI/ARB	25 (5.0)	56 (11.2)	81 (8.1)
Diuretic+CCB+ACE+BB	3 (0.6)	4 (0.8)	7 (0.7)
Diuretic+ACEI/ARB	42 (8.5)	61 (12.2)	103 (10.3)
Diuretic+BB	57 (11.5)	9 (1.8)	66 (6.6)
Other	132 (26.5)	53 (10.6)	185 (18.6)
Miss taking antihypertensive medication 0–1 day per week (good adherence), N (%)	408 (81.3)	452 (90.2)	860 (85.7)
Blood pressure control^, N (%)			
Uncontrolled	382 (78.4)	302 (76.8)	684 (77.7)
Controlled	105 (21.6)	91 (23.2)	196 (22.3)

^*^Data missing for 10 people

^**^Data missing for 3 people; Diuretic typically hydrochlorothiazide; CCB calcium channel blocker, typically amlodipine; *ACEI* angiotensin-converting enzyme inhibitor; *ARB* angiotensin receptor blocker; *BB* beta blocker

^Uncontrolled defined as having either 2/2 or 2/3 of the most recent blood pressures recorded in the prior year ≥ 140 and/or ≥ 90 mm Hg (determined for N = 880 with at least two blood pressure readings)

Respondents had been living with hypertension for a median of 5 years (IQR 3–8) and been on antihypertensive medications for a median of 4 years (IQR 2–7), with no apparent differences by HIV status. Approximately 78% of respondents had uncontrolled blood pressure, with no differences by gender or HIV status. PLHIV self-reported higher adherence to their antihypertensive medications, with 90% reporting missing medication(s) one day per week or less, as compared to 81% of individuals with hypertension only. The most common antihypertensive regimen was a thiazide diuretic (36% overall), and this regimen was more common in PLHIV (44%) compared to those with hypertension only (28%); this was followed by a diuretic plus a calcium channel blocker (typically amlodipine) in 19% of both PLHIV and those with hypertension only. Comorbidity with other NCDs was more common among respondents without HIV: 24% had diabetes compared to 9% of PLHIV, and 4% self-reported heart disease compared to 2% of PLHIV. Individuals living with HIV had been on ART for a median of 11 years (IQR 6–15), and nearly all (96.6%, n = 486) were on first-line ART with tenofovir disoproxil fumarate/lamivudine/dolutegravir (TLD) and were virally suppressed (98.3% of 461 individuals with a result recorded were < 200 copies/mL).

Table 2 shows characteristics of current care. Significant differences were seen in frequency of visits for hypertension care by HIV status, with the majority (60%) of PLHIV attending visits approximately every three months, and only 12% attending visits every one or two months; whereas the majority of individuals with hypertension only (60%) reported monthly or more frequent clinic visits, followed by 37% attending clinic every two months. Respondents spent on average about an hour traveling to receive care, with 85% of PLHIV reporting incurring travel costs as compared to 55% of those with hypertension only (p < 0.001). Individuals with hypertension (without HIV) spent an average of three hours at clinic versus two hours for PLHIV (p < 0.001). Payment for hypertension care (consultation, laboratory testing, or other monitoring tests) was uncommon, reported by 11% of people with hypertension only and 1% of PLHIV (p < 0.001). Similarly, 7% of people with hypertension (without HIV) reported paying for antihypertensive medications as compared to 2% of PLHIV (p < 0.001).

**Table 2:** Characteristics of current hypertension care overall and stratified by HIV status

Characteristics of current care	Hypertension N = 502	Hypertension and HIV N = 501	Overall N = 1003	P-value
Median minutes spent traveling to facility for hypertension care (IQR)	60 (40–120)	60 (45–90)	60 (40–90)	0.21
Median minutes spent at clinic for hypertension care (IQR)	180 (120–240)	120 (60–150)	120 (90–180)	< 0.001
Pay for transportation to clinic	277 (55.2)	426 (85.0)	703 (70.1)	< 0.001
Median USD spent traveling to clinic one way (IQR)*	1.23 (0.61–1.84)	1.23 (0.74–1.84)	1.23 (0.61–1.84)	0.13
Pay for hypertension care^	57 (11.4)	6 (1.2)	63 (6.3)	< 0.001
Median USD spent on hypertension care^ (IQR)*	0.86 (0.86–0.86)	0.74 (0.61–0.86)	0.86 (0.86–0.86)	0.58
Pay for antihypertensive medication(s)	33 (6.6)	8 (1.6)	41 (4.1)	< 0.001
Median USD spent on antihypertensive medication(s)^%^ (IQR)*	4.29 (3.07–7.36)	3.07 (0.43–4.05)	3.87 (3.07–6.13)	0.28
Frequency of visits for hypertension care				
Monthly	299 (59.6)	36 (7.2)	335 (33.4)	< 0.001
Every 2 months	185 (36.9)	22 (4.4)	207 (20.6)	
Every 3 months	18 (3.6)	301 (60.1)	319 (31.8)	
Every 4 months	0 (0)	67 (13.4)	67 (6.7)	
Every 6 months	0 (0)	75 (15.0)	75 (7.5)	
Make extra refill only visits for antihypertensive medications	9 (1.8)	7 (1.4)	16 (1.6)	0.62

^*^Malawi kwacha converted to USD using historical exchange rates using the mid-point of the period of data collection

^Amount paid for a consultation fee and labs or other testing related to hypertension care (excludes costs of medication)

^%^Amount per refill based on current antihypertensive medications

### DCE Results

**Fig. 2:**
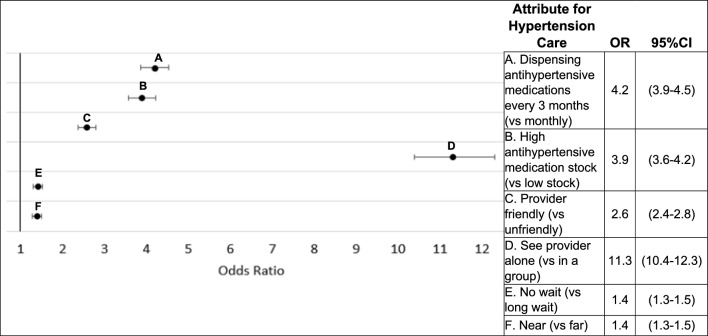
Odds ratios (OR) for DCE attributes

Figure 2 displays the results of the main effects mixed effects logit analysis, assessing preferences among the entire sample. For each attribute, the directionality of preferences was as expected—a nearby facility was preferred relative to a further facility, a friendly provider preferred to an unfriendly one, etc. Overall, the most significant preference was for seeing a provider alone versus in a group (OR 11.3; 95% CI 10.4–12.3). This was followed by a preference for attending clinic for care and medication dispensing every three months (versus monthly) (OR 4.2; 95% CI 3.9–4.5), preference for a facility with high stock of medications (versus low stock) (OR 3.9; 95% CI 3.6–4.2), and preference for a friendly provider (versus unfriendly) (OR 2.6; 95% CI 2.4–2.8). Respondents preferred a facility with no wait time (OR 1.4; 95% CI 1.3–1.5) and that is nearby (OR 1.4; 95% CI 1.3–1.5), although these preferences were less important relative to the other attributes included in the DCE.

Interaction/stratified models were utilized to assess how preferences varied by participant characteristics (Table 3). PLHIV had a significantly stronger preference to see a provider alone rather than in a group as compared to respondents with hypertension only (OR 15.4 versus 8.6, p < 0.001). Conversely, facility wait time and provider friendliness were relatively less important to PLHIV than for individuals with hypertension only (wait time: OR 1.2 versus 1.6, p = 0.001; provider friendliness: OR 2.3 versus 2.9, p = 0.002), although differences were small. Gender differences were seen for several attributes. High stock was more strongly preferred by men than women (OR 4.6 versus 3.5, p = 0.001) while visits every three months were more strongly preferred by women than men (OR 4.7 versus 3.6, p = 0.002). Men had a slightly stronger preference for friendly providers than women (OR 2.9 versus 2.4, p = 0.011). Adults 55 years or older had stronger preferences than younger adults for a facility to be nearby (OR 1.6 versus 1.2, p < 0.001) and for a friendly provider (OR 2.8 versus 2.3, p = 0.04).

**Table 3:** Preferences for DCE attributes stratified by HIV status, gender, and age

Hypertension care attribute	HIV and hyper-tension OR (95%CI)	Hypertension only OR (95%CI)	P-value	Female OR (95%CI)	Male OR (95%CI)	P-value	< 55 years OR (95%CI)	≥ 55 years OR (95%CI)	P-value
Distance: near (vs far)	1.3 (1.1–1.4)	1.5 (1.4–1.7)	0.03	1.4 (1.3–1.6)	1.3 (1.2–1.5)	0.52	1.2 (1.0–1.3)	1.6 (1.4–1.7)	< 0.001
Wait time: no wait (vs long wait)	1.2 (1.1–1.4)	1.6 (1.4–1.8)	0.001	1.4 (1.3–1.5)	1.4 (1.3–1.6)	0.62	1.4 (1.2–1.6)	1.4 (1.3–1.6)	0.89
See provider alone (vs in a group)	15.4 (13.6–17.4)	8.6 (7.6–9.6)	< 0.001	11.7 (10.5–13.1)	11.0 (9.6–12.5)	0.44	12.3 (10.8–14.0)	10.7 (9.6–12.0)	0.12
Friendly provider (vs unfriendly)	2.3 (2.0–2.5)	2.9 (2.6–3.3)	0.002	2.4 (2.1–2.6)	2.9 (2.6–3.3)	0.01	2.3 (2.1–2.6)	2.8 (2.5–3.1)	0.04
Antihypertensive medication stock high (vs low stock)	3.8 (3.3–4.2)	4.1 (3.7–4.6)	0.31	3.5 (3.1–3.8)	4.6 (4.0–5.2)	< 0.001	4.1 (3.7–4.7)	3.7 (3.4–4.1)	0.21
Dispensing antihypertensive medications every three months (3MMD) (vs monthly)	4.7 (4.1–5.3)	3.8 (3.4–4.3)	0.02	4.7 (4.2–5.2)	3.6 (3.2–4.1)	0.002	4.1 (3.6–4.7)	4.3 (3.8–4.8)	0.68

*OR* Odds ratio

### Trade-off Scenarios

**Fig. 3:**
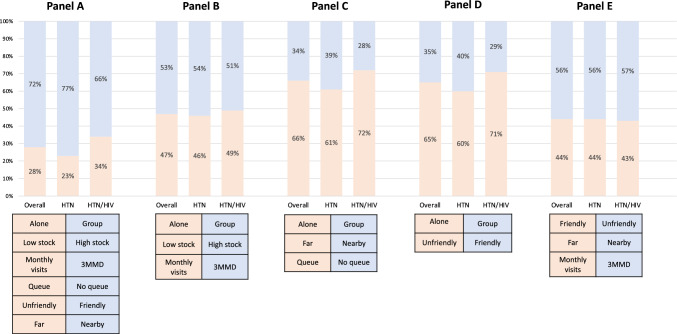
Simulated uptake scenarios overall and stratified by HIV status (N = 1003)

Figure 3 shows scenarios that were simulated to assess individuals’ predicted choices when faced with trade-offs between attributes. While seeing a provider alone was strongly preferred, the majority (72%) of respondents would choose a facility where they saw a provider in a group if the facility had a high stock of hypertension medications, offered three-monthly dispensing, had short wait times, was nearby, and had a friendly provider (Fig. 3, Panel A). PLHIV were less likely to make this trade-off, with an estimated 66% choosing the group model as compared to 77% of individuals with hypertension only. Sixty-six percent of individuals were willing to travel further and wait in a queue in order to see a provider alone (Fig. 3, Panel C); and a greater proportion of PLHIV than those with hypertension only were willing to make this trade-off (72% versus 61%). Similarly, most respondents would choose an unfriendly provider if they saw the provider alone over seeing a friendly provider in a group (65% versus 35%; Fig. 3, Panel D), and a higher proportion of PLHIV were predicted to make this trade-off (71% versus 60%). In several of the assessed scenarios, there was no clear dominant trade-off, with about half of individuals predicted to choose each of the two options (Fig. 2, Panels B and E). Across the five simulated scenarios, there was minimal variation by gender and age (maximum difference in predicted uptake of five percentage points) (data not shown).

## Discussion

Our results suggest a strong preference for individual (versus group) care regardless of HIV status, with willingness to trade-off other attributes to be seen alone with a clinician. These results are somewhat surprising given the expansion of models of group care for HIV, including adherence clubs [27, 28] and community-based ART models [7, 29], the latter being an increasingly accepted and utilized strategy for HIV care delivery throughout Africa. It is possible that this strong preference against group care, particularly among PLHIV, may reflect concern about disclosure if a group were to be comprised of individuals with and without HIV; the DCE scenario did not specify this. Group care models, either facility or community-based, hold promise as a way to overcome limited resources – particularly if counseling and education can be delivered to a large number of people at once. However, group care models for both HIV and/or NCDs may be limited to certain circumstances. For example, Teen Clubs for HIV care have been highly successful in Malawi and provide a much-needed peer community for adolescents living with HIV [30, 31]. Group hypertension care models might have utility for clients with controlled hypertension on a first-line regimen but could be unrealistic for those with uncontrolled hypertension who require frequent medication adjustments and/or older individuals with hypertension-related complications.

Respondents in our study had a strong preference for three-monthly multi-month dispensing (MMD) of antihypertensive medications. These results are in the context of the recent rapid scale-up of three- and six-monthly MMD for ART in Malawi [6]. MMD for ART is highly acceptable [32–34], has been associated with non-inferior outcomes [35, 36], and reduces health system burden [37]. For clients receiving an ART supply of three or six months, coming back to clinic for hypertension refills every one or two months (if clinically stable) can negate the benefits of MMD for ART, such as reduced burden of care, cost savings related to transportation, and time savings. A potential barrier to MMD for NCDs is the fragile supply chain in Malawi, which often results in antihypertensives being unavailable, and can result in clinicians dispensing a smaller quantity (one month or even less) to avoid straining an already limited supply of medication. NCD supply chain challenges are common in many African countries, resulting from underdeveloped systems for forecasting and procurement; a strained supply chain workforce; and weak financing for NCD medications [38–40]. A recent health facility assessment in Malawi showed that only 16% of primary health clinics had availability of three or more antihypertensive classes, and only 37% of facilities had two classes [41]. These findings are in disparate contrast to the ART supply chain across Africa, which is well-funded [42, 43] and has allowed for consistent availability and adequate buffer stocks to facilitate widespread three- and six-monthly MMD.

Respondents in our study, regardless of HIV status, had a relatively strong preference for friendly providers. Provider friendliness also emerged as a strong preference in a DCE in Zambia focused on individuals returning to HIV care after treatment interruption [44]; and was identified as a dominant preference among women with HIV in Tanzania [45]. In the Zambia study, similar to our data from Malawi, respondents were willing to trade off longer distance to a facility and longer wait times for a friendly provider. Data from Africa shows that harsh or unfriendly treatment by providers serves as a barrier to HIV care engagement (and/or re-engagement), and a friendly clinic environment can be a facilitator for engaging in care and remaining on ART [46, 47]. Further research is needed to understand barriers to the provision of friendly, client-centered care, with testing of interventions and their impact on both providers and clinical outcomes for HIV and NCDs. Data from high-income settings suggest that interventions to improve the delivery of client-centered care can improve job satisfaction among health care providers [48].

Uncontrolled hypertension was very common in our study population, with 78% having two or more elevated blood pressures in the prior year, with no difference by HIV status. Our previous work has demonstrated a high rate of uncontrolled blood pressure in PLHIV (80%) [49], with echocardiographic evidence of left ventricular hypertrophy in approximately one-quarter of individuals with uncontrolled blood pressure [50]. Hypertension is a leading contributor to heart disease in Africa [51] and cardiovascular disease risk is higher for PLHIV [52], with data suggesting up to two-fold risk relative to the general population [53, 54]. Therefore, strategies to address the syndemic of HIV and hypertension will be critical for reducing cardiovascular morbidity and mortality in Africa.

This study has several limitations. Because the Ministry of Health aims to provide integrated care for PLHIV in government facilities where hypertension care and medications are delivered for free, we asked respondents with HIV to assume that their care was integrated and that they would receive their HIV and hypertension care (or hypertension only care) at no cost. Therefore, we cannot report on the extent to which PLHIV do or do not prefer integrated care, nor how preferences and trade-offs would change if hypertension care and/or medications were associated with out-of-pocket costs. The majority of PLHIV in this study were enrolled from a subset of clinics that were urban and provide integrated HIV and hypertension care, and all participants enrolled were actively engaged in hypertension care. Therefore, our results may not be generalizable to PLHIV with hypertension in rural settings as well as individuals not engaged in care. These represent important areas for further research.

## Conclusions

Individuals in our study reported strong preferences for individualized rather than group care, three-monthly dispensing of hypertension medications, facilities with adequate antihypertensive medication stock, and friendly providers. While there was some variation in strength of preferences, these results were consistent by HIV status, gender, and age. As Malawi and similar countries strive to scale integrated HIV-NCD care for PLHIV and to improve the delivery of care for all people with NCDs (regardless of HIV status), strategies to strengthen the supply chain to improve availability of hypertension medications and to expand MMD for hypertension and other NCDs will be important for the ability to provide client-centered care. Given high rates of elevated blood pressure in this cohort, understanding how different models of care delivery might improve hypertension control will be critical for improving health outcomes.
